# Beneficial effects and safety of traditional Chinese medicine for chronic inflammatory demyelinating polyradiculoneuropathy: A case report and literature review

**DOI:** 10.3389/fneur.2023.1126444

**Published:** 2023-04-06

**Authors:** Yao Xie, Lesang Li, Le Xie, Junlin Jiang, Ting Yao, Guo Mao, Shiliang Wang, Anchao Lin, Jinwen Ge, Dahua Wu

**Affiliations:** ^1^Department of Neurology, Hunan Hospital of Integrated Traditional Chinese and Western Medicine, Changsha, China; ^2^Ophthalmology Department, Hunan Want Want Hospital, Changsha, China; ^3^Office of Academic Research, Hunan Hospital of Integrated Traditional Chinese and Western Medicine, Changsha, China

**Keywords:** chronic inflammatory demyelinating polyradiculoneuropathy, traditional Chinese medicine, case report, review, *Astragalus membranaceus*

## Abstract

Chronic inflammatory demyelinating polyradiculoneuropathy (CIDP) is an immune-mediated neuropathy. First-line treatments for CIDP include corticosteroids, intravenous immunoglobulin, and plasma exchange. However, the application is always limited by high costs, effectiveness, and adverse events. This study investigated a new potentially effective and safe therapeutic treatment to alleviate CIDP symptoms and improve the quality of life. In the present case, a 47-year-old rural woman presented with weakness and numbness of progressive extremities. She was diagnosed with CIDP based on abnormal cerebrospinal fluid and electromyography. The patient was treated with intravenous dexamethasone for 1 week and with Huangqi-Guizhi-Wuwu and Bu-Yang-Huan-Wu decoctions for 90 days. Surprisingly, after the treatment, the weakness and numbness were eliminated, and the quality of life improved. The varying INCAT, MRC, and BI scores also reflected the treatment effects. After 8 months of discharge, the symptoms did not relapse during the follow-up. We also searched “traditional Chinese medicine (TCM)” and “CIDP” in PubMed, EMBASE, the Web of Science, the Cochrane Library, the Chinese National Knowledge Infrastructure Databases, Wanfang Data, and the Chongqing Chinese Science and Technology Periodical Database. Finally, only ten studies were included in the literature review. Three studies were randomized controlled trials, and seven were case reports or case series. There were 419 CIDP patients, but all study sites were in China. Nine TCM formulas involving 44 herbs were reported, with Huang Qi (*Astragalus membranaceus*) being the most important herb. In conclusion, the case and literature demonstrated that TCM treatment might be a more effective, low-cost, and safe option for treating CIDP. Although these preliminary findings are promising, a larger sample size and higher-quality randomized clinical trials are urgently required to confirm our findings.

## Introduction

Chronic inflammatory demyelinating polyradiculoneuropathy (CIDP) is a rare immune-mediated neuropathy in which an abnormal immune response causes peripheral nerve demyelination and axonal damage (1). It usually results in progressive weakness of the extremities with sensory dysfunction. Symptoms should appear for at least 2 months, and the course can be progressive or relapsing (2). The annual cost of treating CIDP illness can reach USD$ 116,330 per patient, and patients may also experience physical and psychosocial burdens such as impaired physical function, pain, depression, and adverse events (3).

The guidelines recommended intravenous immunoglobulin (IVIg), corticosteroids, and plasma exchange as first-line treatments for CIDP (4, 5). However, the potential side effects of corticosteroids, such as gastric ulceration, diabetes, arterial hypertension, osteoporosis, avascular necrosis of long bones, and cataracts, may outweigh the benefits. Moreover, long-term corticosteroid treatment may cause significant side effects (6). IVIg may result in rapid improvement in disability, but the benefit requires long-term repeated infusions, which carry a significant economic burden, particularly in developing countries (7, 8). Plasma exchange necessitates good vascular access and specialized equipment.

Although first-line treatments can improve symptoms in some patients, they also require ongoing maintenance therapy and the patient's willingness to tolerate the adverse effects or financial burden. The International CIDP Outcome Study (ICOS) revealed that only one-third of CIDP patients were in remission 1 year after the start of first-line treatment, and residual symptoms and deficits were common regardless of treatment (9). There is also a lack of formal efficacy evidence for alternative immunomodulatory agents (4, 10). Because of the side effects, high cost, and limited effectiveness of these treatments, there is an urgent need for new, effective, and safe CIDP treatments.

Traditional Chinese medicine (TCM) has a long history of boosting weakness and sensory dysfunction. TCM treatments have been widely used as a supplement to CIDP treatment in China. Many recent clinical trials have found that TCM treatments can improve clinical symptoms and the quality of life in patients with peripheral neuropathy (PN). Furthermore, TCM therapy can also enhance the sensory nerve conduction velocity (SNCV) and motor nerve conduction velocity (MNCV) in PN (11–13). The therapeutic mechanisms of TCM were elucidated by regulating inflammatory responses and repairing nerve injury (14).

However, no English literature reports the clinical efficacy of Chinese herbs for CIDP. In the present study, we attempted to use short-term corticosteroids combined with Chinese herbs on a patient with CIDP. Surprisingly, we identified that this therapeutic regimen completely relieved limb weakness and numbness and improved the quality of life. A literature review for TCM in CIDP was also conducted.

## Case report

This case report was conducted in accordance with the Case Report (CARE) Guidelines (15).

## Clinical history

**Figure 1:**
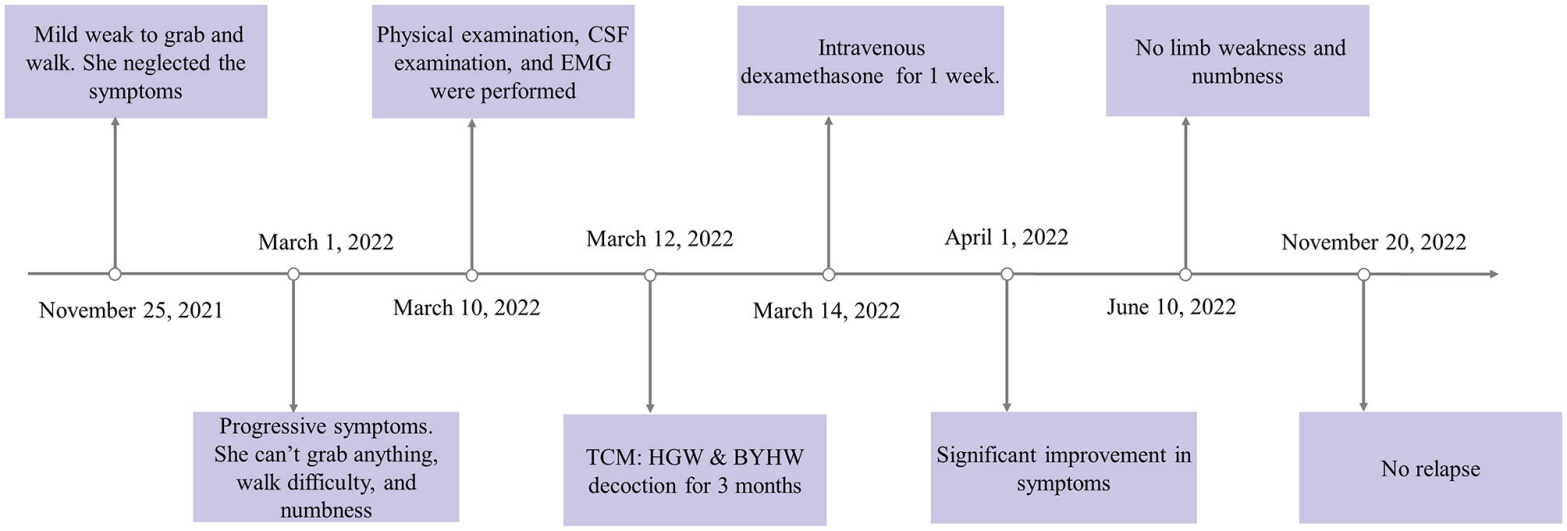
Timeline of the case report. HGW and BYHW decoctions, Huangqi-Guizhi-Wuwu and Bu-Yang-Huan-Wu decoctions; CSF, cerebrospinal fluid; EMG, electromyography.

On 25 November 2021, a 47-year-old rural woman complained of progressive weakness and numbness in her extremities. Initially, her symptoms were mild, causing her to experience difficulty grabbing objects or walking. After 3 months, the symptoms gradually worsened; she could not hold anything and only walked with assistance. On 23 February 2022, she was diagnosed with PN at another hospital. However, no IVIg or corticosteroids were administered to her, and her symptoms did not improve. Then, on 10 March 2022, she was transferred to our hospital (Hunan Hospital of Integrated Traditional Chinese and Western Medicine, Changsha, China). Upon arrival, the woman exhibited signs of both fatigue and limb paralysis, and she specifically emphasized that the numbness she was experiencing felt like she was wearing gloves and socks. She had no other particular medical history (Figure 1).

## Clinical and laboratory examinations

The proximal end of the upper and lower limbs had grade 3 muscle strength, while the distal end of all limbs had grade 2 muscle strength. Muscular atrophy was observed in the bilateral first dorsal interosseous. Tendon reflexes decreased or disappeared, and limb muscular tension also decreased. Pain and temperature sensibility on the distal limbs decreased. Pyramidal tract signs were not elicited. There was no problem with eye montraosvement, pupil size, or swallowing function.

The cerebrospinal fluid (CSF) result revealed that the white blood cell count was one, and the micro-amount of protein was 652.6 mg/L (normal reference value: 150–450 mg/L). Other anti-ganglioside antibodies in CSF and serum were all negative, except for anti-sulfatide (aSF) IgG in CSF. The erythrocyte sedimentation rate (ESR) was 36 mm/h (normal reference value: 0–20 mm/h). Antinuclear antibodies, rheumatoid factors, HBsAg, HCV-Ab, rapid plasma regain, and HIV-Ab, were all negative. The routine blood, stool, and urine examinations, renal and liver functions, myocardial enzymes, and thyroid functions were all normal.

## Electromyography and neuroimaging

Multiple nerves in the upper and lower limbs were observed with decreased MNCV, SNCV, and amplitude during an electromyography (EMG) examination. Demyelination and axonal damage were identified in the bilateral median, ulnar, tibial, and peroneal nerves. Furthermore, no waveforms were elicited from the left peroneal motor nerve, the left median sensory nerve, or the bilateral superficial peroneal sensory nerves (Supplementary Table S1). The brain and spinal MRIs revealed no noticeable abnormalities.

## Diagnosis and treatment

Over 8 weeks, the patient developed progressive, symmetric upper- and lower-limb weakness and numbness and reduced tendon reflexes in all limbs. In addition, abnormal CSF and EMG were observed. The patient was diagnosed with CIDP using the EFNS/PNS consensus guideline (4).

Subsequently, intravenous dexamethasone (20 mg daily) was used for 1 week. Simultaneously, the patient was given TCM. The TCM formulas used in this case report were Huangqi-Guizhi-Wuwu and Bu-Yang-Huan-Wu decoctions (HGW and BYHW), which is composed of 12 herbs: Huang Qi (*Astragalus membranaceus*), 60 g; guizhi (Cassia Twig), 10 g; chishao (Radix Paeoniae Rubra), 10 g; danggui (*Angelica sinensis*), 10 g; chuangxiong (*Ligusticum wallichii*), 10 g; dilong (*Lumbricus*), 10 g; weilingxian (Radix Clematidis), 10 g; fuling (Poria Cocos), 10 g; baizhu (Rhizoma Atractylodis macrocephalae), 10 g; dangshen (*Codonopsis pilosula*), 10 g; Roucongrong (Cistanche), 15 g; and gancao (liquorice), 6 g. The quality of these herbs was consistent with the 2020 Chinese Pharmacopeia (16). The decoction was prepared with a standardized procedure, with each formula unit yielding 400 ml of decoction. The HGW and BYHW decoctions were prescribed for 90 days (200 ml orally two times daily).

## Follow-up and assessment

**Figure 2:**
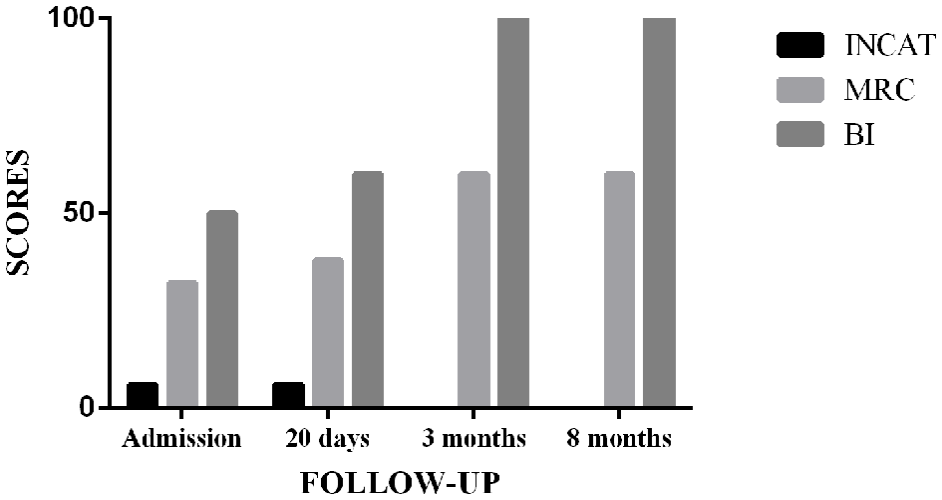
Outcome assessment for the CIDP patient in the follow-up. INCAT, Inflammatory Neuropathy Cause and Treatment; MRC, Medical Research Council; BI, Barthel Index.

Upon admission, the Inflammatory Neuropathy Cause and Treatment (INCAT) Disability Score was 6. The Medical Research Council (MRC) score was 32. The Barthel Index (BI) was 50. After 20 days of treatment, the MRC and BI scores increased to 38 and 60, respectively. However, the INCAT score remained unchanged. Moreover, 3 months later, the patient reported no limb weakness or numbness, and his limb muscle strength could reach grade 5. INCAT, MRC, and BI scores changed to 0, 60, and 100, respectively (Figure 2). After eight months of discharge, the symptoms did not recur during the follow-up.

Most importantly, no side effects were reported. It was unfortunate that she refused to accept an EMG to assess nerve recovery. The patient was delighted with the treatment outcome and was free of adverse events, and the cost was acceptable for a rural family in a developed country. She stated that, after coming to our hospital, her symptoms improved and her quality of life significantly improved. All follow-ups were conducted face to face.

## Literature review

From their inception to 11 November 2022, we screened the following databases for literature reviews: PubMed, EMBASE, Web of Science, the Cochrane Library, the Chinese National Knowledge Infrastructure Databases (CNKI), Wanfang Data, and the Chongqing Chinese Science and Technology Periodical Database (VIP). The search strategy included “traditional Chinese medicine” and “chronic inflammatory demyelinating polyradiculoneuropathy” (Supplementary Table S2).

**Figure 3:**
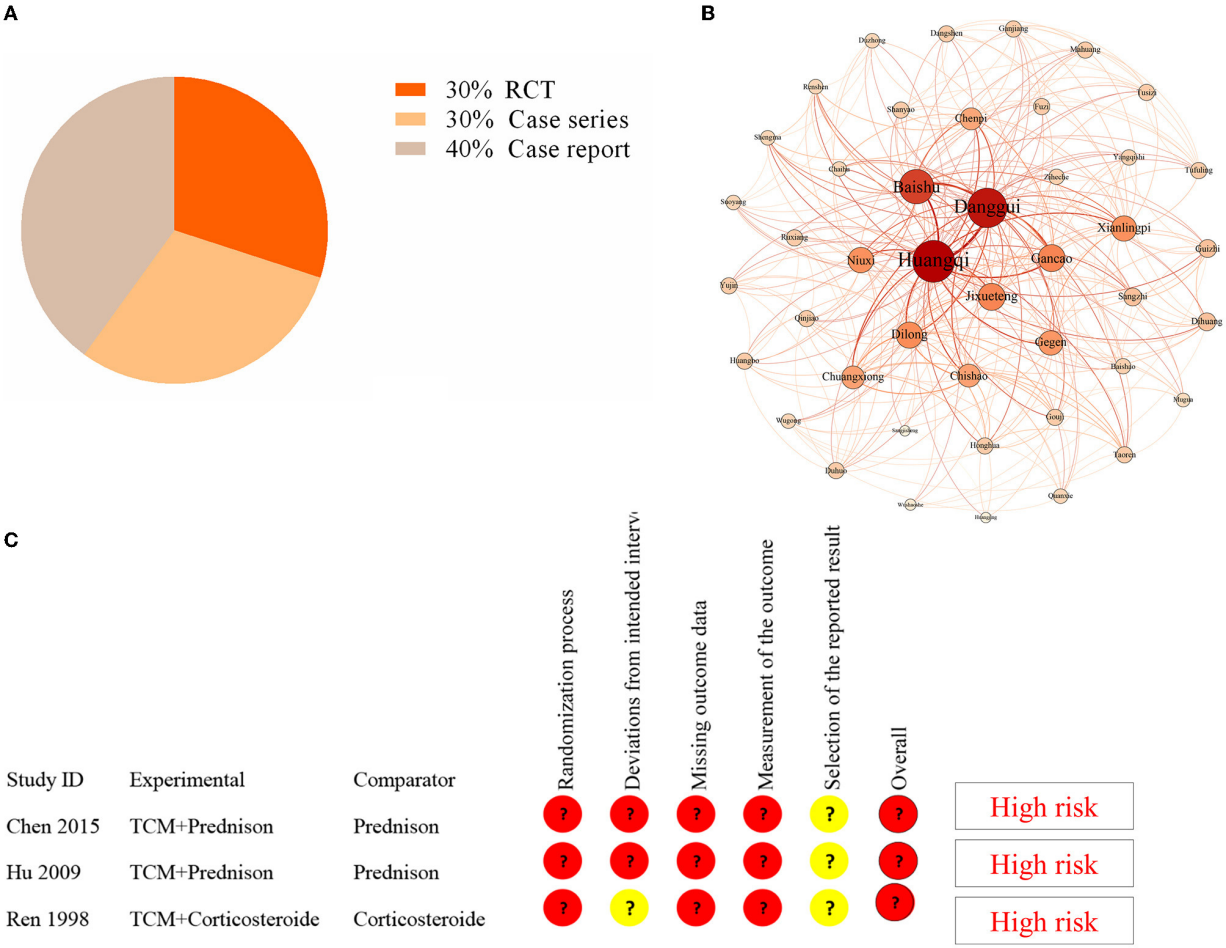
Analysis in the literature review. **(A)** The distribution of study design in the included studies. **(B)** Complex network analysis of selected TCM formulas from the included studies. **(C)** The results of the quality assessment of three RCT studies by using the risk bias assessment tool (RoB2).

Supplementary Figure S1 depicts the selection of studies. Finally, only 10 studies were included in the literature review (Table 1) (17–26). Three studies were randomized controlled trials, while seven were case reports or case series (Figure 3). The earliest study was conducted in 1998. A total of 419 CIDP patients were involved, but all study sites were in China. The age of CIPD cases ranged from 6 to 70 years, with the most enrolled patients being men. The duration of CIDP disease was 2.5 months to 3 years. Most studies did not report comorbidities.

**Table 1:** Study characteristics.

**References**	**Country**	**Cases(*n*)**	**Age**	**Sex (M/F)**	**Course**	**Treatment**	**Significant findings**	**Follow-up**	**Study design**
Sha et al. (17)	China	1	6	Girl	3 years	➢ Huangqi-Guizhi-Wuwu decoction: Huangqi 60 g; Guizhi 10 g; Baishao 15 g; Danggui 15 g; Baizhu 20 g; Gegen 10 g; Sangzhi 6 g; Yingyanghuo 6 g; Yangqishi 6 g; Ziheche 3 g; Dihuang 20 g; Gancao 15 g. ➢ Acupuncture ➢ Course of treatment: 3 months	Hughes: 3–0; Barthel Index: 20–95; muscle strength (upper limbs): 4–5; muscle strength (lower limbs): 3–5	16 months	Case report
Jinliang et al. (18)	China	T: 58 C: 57	T: 37.1 C: 34.7	T: 23/31 C: 28/32	T: 17.1 months C: 15.2 months	➢ T: • Guilongtongluo capsule: Huangqi, Dihuang, Jixueteng, Guizhi, Sangzhi, Dilong, Wushaoshe, etc. Six capsules (0.38 g) tid • Prednison, 15 mg, qd ➢ C: Prednison, 15 mg, qd ➢ Course of treatment: 3 months	MCV/SCV/latency were improved Hughes: Motor T/C: 1.23 ± 1.22/2.13 ± 1.32; Sensory T/C: 0.59 ± 1.02/1.02 ± 1.28; Barthel Index ≤ 40 (%): T/C: 6.9%/21.1%	3 months	RCT
Kuang and Liu (19)	China	1	14	Boy	3 months	➢ Buzhongyiqi-mahuangfuzixixin decoction: Huangqi 30 g; Fuzi 30 g; Dangsheng 15 g; Xianlingpi 15 g; Tufuling 15 g; Shangyao 20 g; Chenpi 9 g; Tusizi 9 g; Baizhu 10 g; Danggui 10 g; Ganjiang 10 g; Jixueteng 45 g; Mahuang 5 g ➢ Maqianzi 2 capsules (0.2 g), bid ➢ Course of decoction: 6 months ➢ Huangqi injection, 40 ml, ivgtt, qd; and shenfu injection, 30 ml, ivgtt, qd ➢ Acupuncture ➢ Course of other TCM: 1 month	Clinical symptoms were full-recovery. Muscular strength and tendon reflex all turned to normal	6 months	Case report
Wei et al. (20)	China	18	40 ± 6.3	10/8	1 ± 0.45 year	➢ TCM Decoction: Huangqi 30 g; Huangjing 15 g; Yinyanghuo 15 g; Sangjisheng 30 g; Niuxi 15 g; Gegen 15 g ➢ Acupuncture and tuina ➢ Course of treatment: 1 month No immunotherapy	Prineas (≥1): 100% to 33.3%; ADL (independence): 0 to 78%	6 months to 12 months	Case series
Yu et al. (21)	China	7	55.8 ± 5.05	4/3	3–26 months	➢ Bu-Yang-Huan-Wu decoction: Huangqi 60 g; Danggui 10 g; Chuangxiong 10 g; Chishao 20 g; Dilong 15 g; Qinjiao 50 g; Baizhu 15 g; Duhuo 15 g; Niuxi 15 g; Jixueteng 30 g; Yujin 15 g; Ruxiang 5 g; Huangbo 10 g ➢ Acupuncture ➢ Course of TCM: 1 month ➢ Prednosone	Muscular strength increased (≥2) 71.43%; (≥1) 100%	Not report	Case series
Liu et al. (22)	China	1	33	Woman	1 year	➢ Buzhongyiqi decocotion: Huangqi 30 g; Renshen 10 g; Baizhu 10 g; Danggui 10 g; Shengma 10 g; Chaihu 10 g; Chenpi 10 g; Duzhong 10 g; Niuxi 10 g; Suoyang 10 g; Gancao 6 g ➢ Acupuncture and moxibusion ➢ Course of TCM: 1 month ➢ Corticosteroid	Muscular strength was from 2+ to 5–; Numbness and tendon reflex all turned to normal	Not report	Case report
Hu et al. (23)	China	T: 30 C: 30	T: 35.8± 5.2 C: 36.6± 7.3	T: 18/12 C:20/10	T:11.1 ± 4.5 months C:10.3 ± 4.8 months	➢ T: • Guilongtongluo capsule: Huangqi, Dihuang, Jixueteng, Guizhi, Sangzhi, Dilong, Wushaoshe, etc. Six capsules (0.38 g) tid • Prednison, 15 mg, qd ➢ C: Prednison, 15 mg, qd ➢ Course of treatment: 3 months	MCV/SCV/latency were improved Hughes: Motor T/C: 1.27 ± 1.25/2.06 ± 1.43; Sensory T/C: 0.63 ± 0.93/1.28 ± 1.37; Barthel Index ≤ 40 (%): T/C: 10%/20%	3 months	RCT
Li and Fan (24)	China	15	20–70	10/5		➢ Bu-Yang-Huan-Wu decoction: Huangqi 30 g; Danggui 12 g; Chuangxiong 9 g; Chishao 12 g; Dilong 12 g; Taoren 12 g; Gouji 20 g; Gegen 20 g; Honghua 9 g; Quanxie 9 g, 2 Wugong ➢ Course of treatment: 4 weeks	Prineas (≥1): 100% to 26.7%	6 months	Case series
Lin et al. (25)	China	11	11–65	6/5	1.5 years	➢ Bu-Yang-Huan-Wu decoction: Huangqi 45 g; Danggui 12 g; Chuangxiong 9 g; Chishao 12 g; Dilong 12 g; Taoren 12 g; Gouji 15 g; Mugua 6 g; Honghua 5 g; Gancao 5 g ➢ Course of treatment: 6 months ➢ Intravenous inmmunoglobulin 0.4 mg/kg, ivgtt, qd, 5 days	Prineas (≥1): 100% to 9.1%	6 months−12 months	Case series
Ren et al. (26)	China	T: 20 C: 20	T: 35 ± 2 C: 33 ± 3	T: 12/8 C: 11/9	T: 3–8 months C: 2.5–7 months	➢ T: • Buzhongyiqi decocotion: Huangqi 30 g; Renshen 15 g; Baizhu 10 g; Danggui 10 g; Shengma 10 g; Chaihu 10 g; Chenpi 10 g; Gancao 6 g • Corticosteroid ➢ C: Corticosteroid ➢ Course of treatment: 3 months	Muscular strength increased (≥2) 65%/30%; (≥1) 90%/70%; Relapse rate: T/C: 11.1%/42.9%	12 months	RCT

TCM decoctions were used in all included studies, and the treatment duration ranged from 4 weeks to 6 months. In 60% of the studies, TCM was combined with corticosteroids or IVIg. The average duration of follow-up ranged from 6 months to 16 months. TCM therapies improved muscular strength, sensory disorders (SNCV and MNCV), daily life ability, and relapse rate. However, many studies did not report any adverse event.

There have been reports of nine TCM formulas involving 44 herbs (Supplementary Table S3). The most common herbs were Huang Qi (*Astragalus membranaceus*) (100%), Banggui (*Angelica sinensis*) (77.8%), Baizhu (Rhizoma Atractylodis macrocephalae) (44.4%), and Dilong (*Lumbricus*) (44.4%). We used Gephi 0.9.2 and SPSS Modeler 18.0 to perform a complex network analysis to find the core herb. We identified that Huang Qi was the core herb. The results revealed that the number of triangles, clustering coefficient, and eigenvector centrality were 291, 0.32, and 1, respectively (Figure 3). Furthermore, the dose of Huang Qi in various formulas ranged from 30 to 60 g.

Three RCT studies had sample sizes ranging from 40 to 115. The risk bias assessment tool (RoB2) (27) was used to assess the quality of three RCT studies. The randomization process, deviations from the intended intervention, missing outcome data, outcome measurement, and selection of the reported result were all evaluated. However, the bias in these studies indicated a high risk (Figure 3). Therefore, the efficacy of TCM for CIDP requires further validation in a high-quality RCT.

## Discussion

The typical CIDP is a chronic, progressive, monophasic, or recurrent demyelinating polyradiculoneuropathy with progressive weakness, sensory dysfunction, and absent or reduced tendon reflexes (28). CIDP variants include distal, multifocal, focal, motor, and sensory CIDP, all of which share the characteristics of demyelination and the immune therapy response (4). The diagnosis of CIDP is mainly based on clinical, electrodiagnostic, and laboratory features. However, the heterogeneity of CIDP creates challenges and delays in diagnosis. The ICOS study indicated that the time between disease onset and treatment was 11 months, and delayed-treated patients had a more severe disability (9).

The CIDP patient discussed in the above care report presented with symmetrical and progressive weakness, numbness, loss of tendon reflexes, abnormal protein levels in the CSF, and demyelination on the EMG. The progressive phase lasted more than 3 months. Furthermore, this patient responded to corticosteroid treatment, one of the characteristics of CIDP (29). Therefore, there was sufficient evidence to confirm a CIDP diagnosis for the patient. However, treatment was delayed due to economic hardship and initial mild symptoms.

Interestingly, the patient's CSF ASF-IgG was positive. One of the glycolipid antibodies against the peripheral nerve membrane surface is aSF. Sulfatides constitute a significant class of myelin-specific lipids, and their depletion can result in region-specific effects on non-compact myelin (30). Only 1–3% of CIDP patients had positive aSF (31, 32), raising questions about its diagnostic value. However, aSF-positive patients can exhibit typical clinical symptoms and respond well to immunomodulatory therapy (33), and this case supports the conclusion. The pathological mechanism and clinical significance of aSF should be investigated further.

Almost 20% of the CIDP patients did not respond to first-line therapy (5). Furthermore, 85.7% and 76.9% of CIDP patients experienced worsening after discontinuing IVIg and intravenous methylprednisolone, respectively (34). In addition, the long-term treatment course, high costs, inconvenience, and adverse events limit the application. There is a pressing need to explore alternative therapies that are both effective and safe in order to avoid those problems. Some studies have demonstrated that pulsed high-dose corticosteroid treatment might have fewer side effects and a faster response than daily oral corticosteroid treatment (35, 36). However, the evidence in the guideline is only low to moderate (4).

Meanwhile, some researchers have focused on subcutaneous immunoglobulin, which is well tolerated, effective, and affordable. However, the duration of maintenance treatment is at least 24 weeks, and local reactions at the infusion site are common (37). Better therapies must be explored further.

Traditional Chinese medicine and intravenous dexamethasone were used to treat this case. The intravenous dexamethasone course lasted only 1 week, and TCM was used for 3 months. With no adverse events and an extremely positive response, the combined treatment may be a more effective and safe option. The short-term corticosteroid treatment was well tolerated. Moreover, Chinese herbs were inexpensive, had few or no side effects, and were readily available. In the literature review, 50% of the studies (17, 20–22, and 25) used TCM in combination with corticosteroids, resulting in improvements in weakness, sensory disorder, daily life ability, and relapse rate. Therefore, it can potentially solve the CIDP dilemma, particularly in developing and rural countries. It is generally known that CIDP is an autoimmune disorder that affects peripheral nerve components. Macrophage and T-cell infiltration into peripheral nerves or nerve roots can result in demyelination and axonal damage (2). Corticosteroids are believed to treat CIDP by suppressing various immune components of its presumed autoimmune inflammatory process. Higher concentrations of corticosteroids also interact with DNA recognition sites to activate the transcription of anti-inflammatory genes (6).

Traditional Chinese medicine treatment of this reported CIDP case included Huangqi-Guizhi-Wuwu (HGW) and Bu-Yang-Huan-Wu (BYHW) decoctions, which are the most famous formulas in ancient TCM books. The network pharmacology analysis revealed that HGW could regulate cytokines and the inflammatory response, elements of the cytokine network, the T-cell receptor, NF-kappa B, HIF-1, and PI3K-Akt signaling pathways (14). BYHW can also regulate inflammatory cytokine production and facilitate axonal regeneration (38). Moreover, BYHW may assist in the timely interaction of regenerating axons and distal Schwann cells (39). Furthermore, some studies have indicated that quercetin, formononetin, isorhamnetin, and kaempferol were the main active compounds of HGW, while hydroxysafflor yellow A, astragaloside IV, ferulic acid, ligustrazine, and Z-ligustilide were mainly present in BYHW (40). In the literature review, the herb Huang Qi was used in all studies, with doses ranging from 30 to 60 g in different formulas, and it was used as the core herb in the complex network analysis. This female CIDP patient was also treated with 60 g Huang Qi. More than 100 compounds have been identified in Huang Qi, including flavonoids, saponins, polysaccharides, and amino acids (41). Huangqi can promote the development of immune organs, enhance mucosal immune function, increase the quantity and phagocytic capacity of innate immunity, promote the maturation and differentiation of acquired immunity cells, and improve antibody expression in acquired immunity (42). It appears promising to conduct extensive research on these formulas and Huang Qi for CIDP.

The Inflammatory Neuropathy Cause and Treatment Disability Score, MRC, and BI were used in this case to assess the treatment effect at multiple time points. The INCAT disability score is a valuable tool for evaluating upper and lower limb dysfunctions in inflammatory polyneuropathy studies, with the advantages of feasibility, high face validity, and high reliability (43). The MRC scale was used to assess muscle strength, with a score ranging from 0 for paralysis to 5 for normal and the sum of six pairs of muscles to represent a patient's overall strength. For inflammatory neuropathies, the MRC grading system was frequently used as an outcome measure (44). The BI is a reliable indicator of independence in daily activities. The scale described ten tasks based on the amount of time or assistance the patient requires, with lower scores indicating greater nursing dependency (45, 46). Surprisingly, after 3 months of TCM treatment, the INCAT, MRC, and BI scores were close to normal, and the treatment effect was sustainable. There was no relapse in the 8 months of follow-up.

Adverse effects were common with short-, medium-, and long-term immunotherapy (47). Adverse effects of corticosteroids include diabetes mellitus, hypertension, gastric ulceration, weight gain, osteopenia, and the long-term risk of severe side effects. Transient hypertension, headache, venous thrombosis, and acute renal dysfunction are the common side effects of IVIg. Adverse events associated with citrate use, difficulty with venous access, and hemodynamic changes have been reported in plasma exchange patients. In some cohorts, 60–70% of the CIDP patients reported problems with mobility, self-care, usual activities, pain, or discomfort in IVIg and methylprednisolone (9). It is inspiring to be here and see how TCM can be used to ensure efficacy and safety in the literature review and case report. Reducing adverse events is particularly important for CIDP patients.

To the best of our knowledge, to date, no English literature has reported the clinical efficacy of Chinese herbs for CIDP. This is the first article to summarize TCM trial or case report findings for CIDP. The article also presented potentially effective herbs or formulas containing specific ingredients. However, evidence of efficacy in case reports is lacking. Longer follow-up efficacy and an EMG exam should be investigated further. All the studies in the review were published in Chinese literature, had a single study site, and had low study quality. Large-sample, multi-center, high-quality studies are required to obtain a high level of evidence for TCM. Therefore, TCM has broad potential applications in CIDP because TCM is well tolerated, effective, inexpensive, and readily available.

## Conclusion

In conclusion, the case demonstrated that TCM treatment combined with short-term corticosteroid therapy eliminated the weakness and numbness of CIDP and improved her quality of life. Huangqi (*Astragalus membranaceus*) may be important for regulating the inflammatory response. Although these preliminary findings are promising, larger sample sizes and higher-quality RCTs are urgently required to confirm our findings.

## Data availability statement

The original contributions presented in the study are included in the article/Supplementary material, further inquiries can be directed to the corresponding author.

## Ethics statement

Ethical review and approval was not required for the study on human participants in accordance with the local legislation and institutional requirements. The patients/participants provided their written informed consent to participate in this study. Written informed consent was obtained from the individual(s) for the publication of any potentially identifiable images or data included in this article.

## Author contributions

The idea for this research was proposed by YX. This protocol's drafting was undertaken by YX and LL. DW and JG revised the manuscript for intellectual content. Data collection and illness discussion were performed by YX, LX, JJ, GM, TY, SW, and AL. All authors approved the final version of the manuscript.
