# Supplementary material for: Beneficial effects and safety of traditional Chinese medicine for chronic inflammatory demyelinating polyradiculoneuropathy: A case report and literature review
Source: Front Neurol. 2023 Apr 6;14:1126444. doi: 10.3389/fneur.2023.1126444 (PMC10115958; doi:10.3389/fneur.2023.1126444)
Supplement: Supplementary file 1 [file Data_Sheet_1.DOCX]

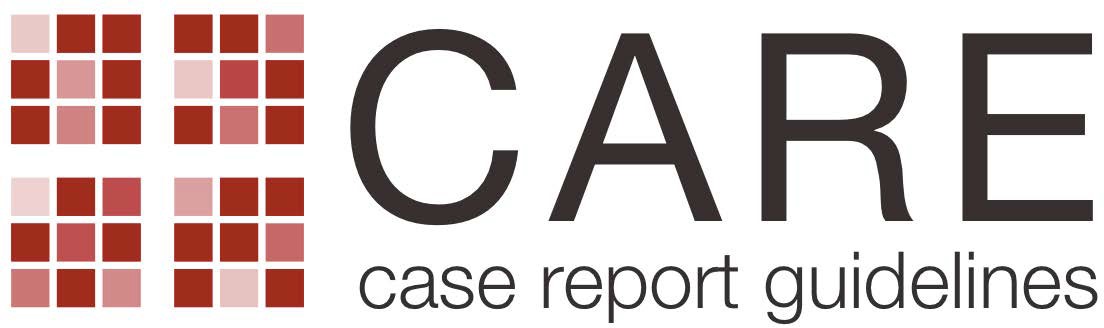
**CARE Checklist of information to include when writing a case report
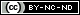
**

**Topic Item Checklist item description Reported on Line**

**Title 1** The diagnosis or intervention of primary focus followed by the words “case report” **Line 1-2**

**Key Words 2** 2 to 5 key words that identify diagnoses or interventions in this case report, including "case report" **Line 15-16**

# Abstract

**(no references)**

**3a** Introduction: What is unique about this case and what does it add to the scientific literature? **Line 18-21**

**3b** Main symptoms and/or important clinical findings **Line 21-24**

**3c** The main diagnoses, therapeutic interventions, and outcomes **Line 24-30**

**3d** Conclusion—What is the main “take-away” lesson(s) from this case? **Line 38-41**

**Introduction 4** One or two paragraphs summarizing why this case is unique (**may include references**) **Line68-80**

**Patient Information 5a** De-identified patient specific information **Line84**

**5b** Primary concerns and symptoms of the patient **Line84-92**

**5c** Medical, family, and psycho-social history including relevant genetic information **Line92-93**

**5d** Relevant past interventions with outcomes **Line80-89**

# Clinical Findings

**Timeline**

**Diagnostic Assessment**

**Therapeutic Intervention**

**Follow-up and Outcomes**

1. Describe significant physical examination (PE) and important clinical findings **Line94-116**
2. Historical and current information from this episode of care organized as a timeline **Line454-456(Figure1 )**

**8a** Diagnostic testing (such as PE, laboratory testing, imaging, surveys). **Line118-121**

**8b** Diagnostic challenges (such as access to testing, financial, or cultural) **Line143**

**8c** Diagnosis (including other diagnoses considered) **Line121-122**

**8d** Prognosis (such as staging in oncology) where applicable --

**9a** Types of therapeutic intervention (such as pharmacologic, surgical, preventive, self-care) **Line123-125**

**9b** Administration of therapeutic intervention (such as dosage, strength, duration) **Line** 123,125-131

**9c** Changes in therapeutic intervention (with rationale) --

**10a** Clinician and patient-assessed outcomes (if available) **Line136-141**

**10b** Important follow-up diagnostic and other test results **Line138-143**

**10c** Intervention adherence and tolerability (How was this assessed?) **Line142-144**

**10d** Adverse and unanticipated events **Line142**

**Discussion 11a** A scientific discussion of the strengths AND limitations associated with this case report **Line276-285**

**11b** Discussion of the relevant medical literature **with references** **Line182-175**

**11c** The scientific rationale for any conclusions (including assessment of possible causes) **Line287-292**

**11d** The primary “take-away” lessons of this case report (without references) in a one paragraph conclusion **Line287-288**

**Patient Perspective 12** The patient should share their perspective in one to two paragraphs on the treatment(s) they received **Line143-147**

**Informed Consent 13** Did the patient give informed consent? Please provide if requested . . . . . . . . . . . . . . . . . . . . . . . . . . . . . . . . . . . . . . **Yes ☑ No**
