# Supplementary material for: Beneficial effects and safety of traditional Chinese medicine for chronic inflammatory demyelinating polyradiculoneuropathy: A case report and literature review
Source: Front Neurol. 2023 Apr 6;14:1126444. doi: 10.3389/fneur.2023.1126444 (PMC10115958; doi:10.3389/fneur.2023.1126444)
Supplement: Supplementary file 2 [file Data_Sheet_2.DOCX]

**Table S1：Electromyogram report**

**Motor nerve conduction velocity measurement**

| **Nerve** | **Latency period**  **(ms)** | **Amplitude**  **(mV)** | **Conduction velocity (m/s)** |
| --- | --- | --- | --- |
| **Left Medianus** | | | |
| Post. 1－Rec pos | 3.2 | 0.0 ↓↓↓ | 37.5 ↓ |
| Post. 2－Post. 1 | 8.0 | 0.1 ↓↓↓ |  |
| **Left Ulnaris** | | | |
| Post. 1－Rec pos | 3.2 | 0.8 ↓↓↓ | 51.2 |
| Post. 2－Post. 1 | 7.3 | 0.6 ↓↓↓ |  |
| **Right Tibialis** | | | |
| Post. 1－Rec pos | 4.1 | 1.9 ↓↓↓ | 47.6 |
| Post. 2－Post. 1 | 10.4 | 1.0 ↓↓↓ |  |
| **Left Tibialis** | | | |
| Post. 1－Rec pos | 4.6 ↑ | 0.4 ↓↓↓ | 42.7 ↓ |
| Post. 2－Post. 1 | 12.1 ↑ | 0.5 ↓↓↓ |  |
| **Right Peroneus** | | | |
| Post. 1－Rec pos | 5.0 ↑ | 0.1 ↓↓↓ | 40.8 ↓ |
| Post. 2－Post. 1 | 12.9 ↑ | 0.1 ↓↓↓ |  |
| **Right Peroneus** | | | |
| Post. 1－Rec pos | **--** | **--** | **--** |
| Post. 2－Post. 1 | **--** | **--** |  |

**Sensory nerve conduction velocity measurement**

| **Nerve** | **Latency period**  **(ms)** | **Amplitude**  **(mV)** | **Conduction velocity (m/s)** |
| --- | --- | --- | --- |
| **Left Medianus** | | | |
| Stim. 1－Rec 1 | **--** | **--** | **--** |
| **Left Ulnaris** | | | |
| Stim. 1－Rec 1 | 3.2 | 3.6 ↓ | 60.9 |
| **Right Peroneus super** | | | |
| Stim. 1－Rec 1 | **--** | **--** | **--** |
| **Right Peroneus super** | | | |
| Stim. 1－Rec 1 | **--** | **--** | **--** |

‘’—‘’represent that the waveform was not elicited.

**Table S2：Search strategy**

| **For example: Pubmed database** |
| --- |
| #1 (((chronic inflammatory demyelinating polyradiculitis[Title/Abstract]) OR (Chronic inflammatory demyelinating polyradiculoneuropathy[Title/Abstract])) OR (CIDP[Title/Abstract]))  #2 ((((((TCM[Title/Abstract]) OR (traditional Chinese medicine[Title/Abstract])) OR (Chinese medicine[Title/Abstract])) OR (Chinese herb[Title/Abstract])) OR (herb[Title/Abstract])) OR (decoction[Title/Abstract]))  #3 #1 AND #2 |

**Table S3：Summary table of herbs in studies**

| **Herb** | **Latin names** | **Frequency** |
| --- | --- | --- |
| Huangqi | Astragalus membranaceus | 9 |
| Danggui | Angelica sinensis | 7 |
| Baizhu | Rhizoma Atractylodis Macrocephalae | 5 |
| Dilong | Lumbricus | 4 |
| Gancao | Liquorice | 4 |
| Niuxi | Radix Achyranthis Bidentatae | 3 |
| Chenpi | Pericarpium Citri Reticulatae | 3 |
| Chishao | Radix Paeoniae Rubra | 3 |
| Chuangxiong | Ligusticum Wallichii | 3 |
| Gegen | Pueraria Lobata | 3 |
| Jixueteng | Caulis Spatholobi | 3 |
| Xianlingpi | Herba Epimedii | 3 |
| Chaihu | Bupleurum Falcatum | 2 |
| Dihuang | Rhizoma Rehmanniae | 2 |
| Gouji | Lycium Barbarum | 2 |
| Guizhi | Cassia Twig | 2 |
| Honghua | Carthamus Tinctorius | 2 |
| Renshen | Ginseng | 2 |
| Sangzhi | Ramulus Mori | 2 |
| Shengma | Rhizoma Cimicifugae | 2 |
| Taoren | Peach Kernel | 2 |
| Ganjiang | Rhizoma Zingiberis | 1 |
| Ruxiang | Olibanum | 1 |
| Sangjisheng | Parasitic Loranthus | 1 |
| Yujin | Radix Curcumae | 1 |
| Baishao | Radix Paeoniae Alba | 1 |
| Dangshen | Codonopsis Pilosula | 1 |
| Duhuo | Radix Angelicae Pubescentis | 1 |
| Duzhong | Eucommia Ulmoides | 1 |
| Ziheche | Placenta Hominis | 1 |
| Fuzi | Radix Aconiti Carmichaeli | 1 |
| Huangbo | Golden Cypress | 1 |
| Huangjing | Rhizoma Polygonati | 1 |
| Mahuang | Chinese Ephedra | 1 |
| Mugua | Fructus Chaenomelis | 1 |
| Qinjiao | Radix Gentianae Macrophyllae | 1 |
| Quanxie | Buthus Martensi Karsch | 1 |
| Shanyao | Chinese Yam | 1 |
| Suoyang | Cynomorium Songaricum Rupr | 1 |
| Tufuling | Rhizoma Smilacis Glabrae | 1 |
| Tusizi | Semen Cuscutae | 1 |
| Wugong | Centipede | 1 |
| Wushaoshe | Zaocys Dhumnade | 1 |
| Yangqishi | Actinolitum | 1 |


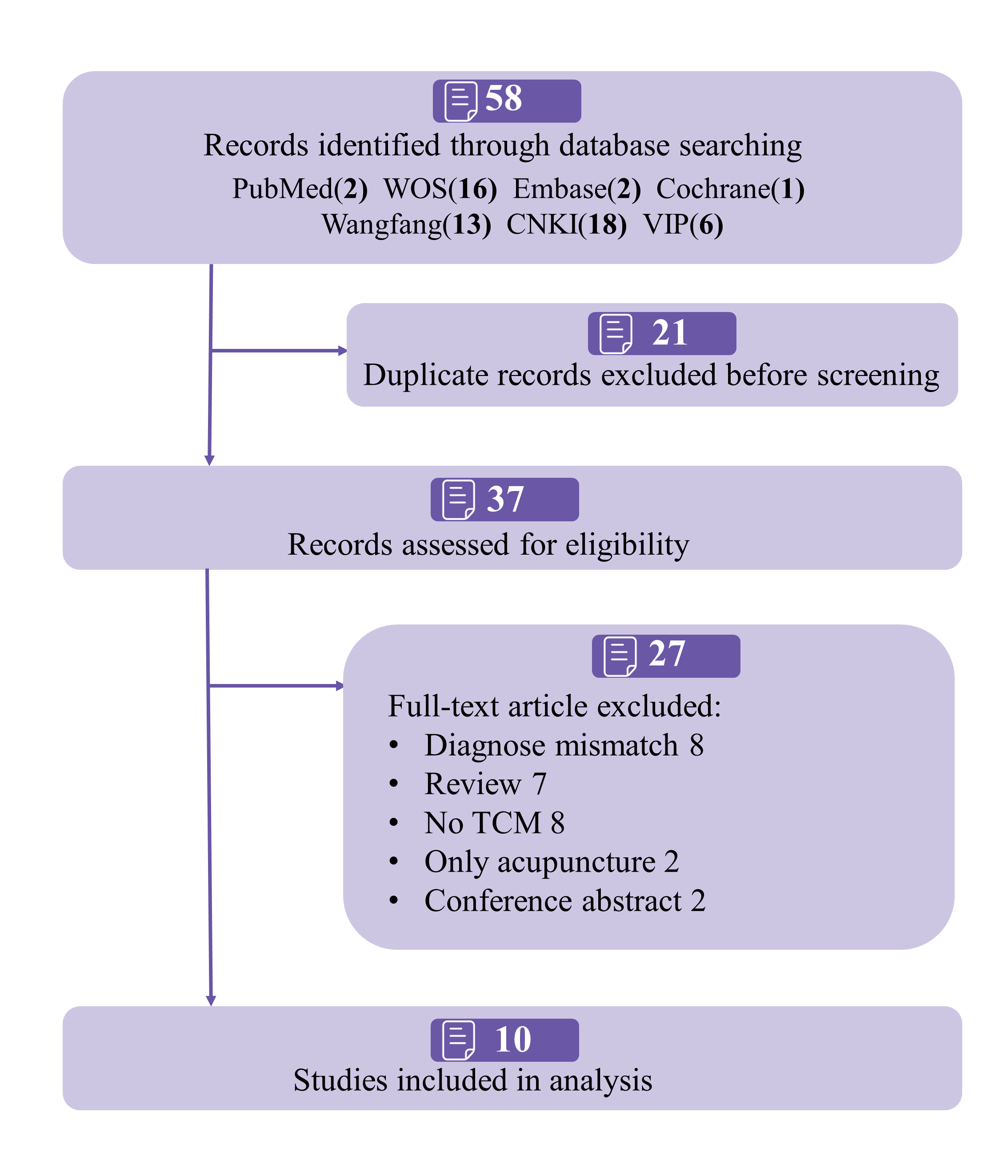


Figure S1: Flowchart of study selection.
